# Determinants of Intention to Purchase Bottled Water Based on Business Online Strategy in China: The Role of Perceived Risk in the Theory of Planned Behavior

**DOI:** 10.3390/ijerph182010729

**Published:** 2021-10-13

**Authors:** Meiwen Guo, Cheng Ling Tan, Liang Wu, Jianping Peng, Rongwei Ren, Chun-Hung Chiu

**Affiliations:** 1 School of Management, Guangzhou Xinhua University, Guangzhou 510520, China; gmw@xhsysu.edu.cn (M.G.); 2 Graduate School of Business, Universiti Sains Malaysia, Penang 11800, Malaysia; 3 Entrepreneurship Center, Sun Yat-sen University, Guangzhou 510275, China; 4 School of Marxism, Sun Yat-sen University, Guangzhou 510275, China; 5 School of Business, Sun Yat-sen University, Guangzhou 510275, China; mnsrrw@mail.sysu.edu.cn (R.R.); zhaojx5@mail.sysu.edu.cn (C.-H.C.)

**Keywords:** online bottled water, risk perception, water pollution, non-degradable packaging, business online strategy, public health, sustainable consumption

## Abstract

With the development of the network economy, especially the promotion and popularization of mobile networks, traditional offline businesses are further integrated with online businesses, promoting the development of business online strategies. However, with the growth of enterprises’ business, their negative externalities on the environment have gradually become prominent, further affecting sustainable consumption. The relationships between businesses, the environment, and consumption have become the focus of attention. China’s fast-growing bottled water companies face similar challenges. The pollution that occurs due to bottled water packaging poses great threats to consumers. Hence, this study extended the Theory of Planned Behavior (TPB) by integrating three risk aspects, namely, water pollution risk perception (WPRP), non-degradable package pollution risk perception (NPPRP), and false information risk perception (FIRP), to examine the consumers’ perceptions toward these risk aspects before purchasing bottled water online. This study employed a cross-sectional approach to collect data from online consumers via a survey method. A total of 401 valid samples were collected and then analyzed via a structural equation model using the AMOS statistical package. The results showed that attitude (AT), subjective norm (SN), and perceived behavior control (PBC) toward online bottled water purchase had significant and positive effects on the consumers’ purchase intentions (PIs). However, under the influence of risk perception, the consumers’ attitudes, SNs and PBC became suppressed by WPRP, and SN became suppressed due to the impact of FIRP. Furthermore, the negative impacts of NPPRP and FIRP on PI were partially mediated by AT, SN and PBC. Meanwhile, WPRP imposed the most significant direct effect on PI. The study results will help businesses to develop better online strategies to reduce the risk perception of bottled water and provide theoretical value and practical guidance for realizing sustainable consumption.

## 1. Introduction

With the rapid development of e-commerce platforms and the long-term impact of COVID-19, online business has been further strengthened, offline businesses have been weakened, and business online strategies have been further deepened and developed. According to the statistics of statistia.com, by 2021, the world’s online transaction volume will reach 4.48 trillion USD, almost twice that of 2017 (2.29 trillion USD). China’s clothing, food, daily necessities and other enterprises are actively building online strategies to enhance their competitiveness and continuously expand their market share. The coronavirus pandemic hit China’s offline consumption in 2020, but online consumption grew against this trend. According to the China National Bureau of Statistics data, in 2020, the annual online retail sales increased by 10.9% year-on-year, while the total retail sales of social consumer goods decreased by 3.9%, forming a huge contrast between the two [1].

Businesses’ online strategies will become more and more mature, which will change the pattern of the retail market and deeply affect consumer behavior [2]. On the one hand, the achievements of enterprises’ online business have promoted more enterprises to allow for open online business, forming a multi-level competition that breaks through space and time; on the other hand, consumers’ offline purchasing behavior is transformed into a consumption experience, where direct consumption is reduced, while they turn to online indirect consumption to obtain goods through logistics distribution. This new consumption mode replaces the traditional consumption mode [3]. While maximizing the profits of upstream supply chain and online business, entity enterprises can only invest more energy in the circulation link connecting production and consumption, that is, constantly optimize the online strategy [4]. Therefore, business online strategies have an important impact on the sustainable development of enterprises. Online strategies provide conditions for marketing innovation and are of great significance in recovering from the global economic crisis brought about by covid-19 [5].

In China, bottled water has experienced more than 70 years of development since it was developed in the 1920s. Now, the bottled water industry has become one of the largest sub-industries of China’s soft drink industry, and the revenue brought by this industry accounts for about 20% of the revenue of China’s soft drink industry. The scale of bottled water will be further expanded in the future. In 2019, China’s per capita consumption of bottled water was only 34 liters, far lower than that of the United States and similar to Japan’s. It is expected that the per capita consumption of bottled water in China will further increase in the future and is expected to exceed that of Japan [6]. From the historical growth rate of China’s per capita consumption of various categories, it can be found that since 2013, the driving force of China’s per capita beverage consumption mainly comes from bottled water. In the future, with the improvement of Chinese residents’ health awareness, the demand for bottled water will grow faster than other sugary soft drinks.

However, compared with other industries, China’s bottled water industry is not conducive to the healthy development of the bottled water market due to its fast development speed, small product differences, lack of or short-sighted enterprise strategy, blind development, and pursuit of short-term interests [7]. With the rapid development of mobile networks, online channels become the focus of bottled water enterprises’ development strategies. It becomes more and more convenient to buy bottled water through the Internet. Consumers can order bottled water through the official website of a bottled water brand via Taobao, Tmall, JD, the WeChat applet, and other platforms.

Scholars have conducted a lot of analysis on the differences and substitutability between bottled water and other water sources [8,9], and the substances contained [10,11], and reached a consensus on the convenience of bottled water and the importance of natural mineral components. However, bottled water also led to a series of environmental problems due to business online strategies causing unsustainable consumption, and there is a lack of consensus on the safety of bottled water [12,13] and its impact on the environment [14,15]. The water sources of bottled water and standards of water are different across countries; therefore, the safety testing of bottled water needs a larger sample. Additionally, the impact of environmental pollution on water sources and the environmental pollution caused by bottled water packaging needs more attention. This is related to the future development of the bottled water market and has a long-term impact on human health. In particular, online strategies have expanded the water market and in return, caused several environmental problems. Thus, in this study, we aimed to essay to address the following research questions.

RQ1: What factors influence online bottled water consumption behavior? How do the mechanisms of these factors work?

PQ2: What are the environmental risks that consumers perceive regarding online bottled water? How do these factors affect consumer behavior?

With the continuous improvement of consumers’ online purchasing experience, bottled water purchasing habits have gradually shifted from offline purchases to online purchases. The water quality of bottled water sources, the sustainability of packaging, and the authenticity of publicity information online have become a matter of keen concern for consumers. First of all, bottled water sources are often in areas with better water quality, and there are many sources of water in ecologically protected areas. However, with the development of surrounding agriculture, industry, and tourism, the use of many chemical fertilizers, industrial wastewater discharge, and tourist waste will lead to water pollution, which will negatively impact the surrounding ecological environment and consumers’ health. Second, if the plastic packaging that is used for bottled water cannot be recycled and processed in time, it will cause environmental pollution. In addition, harmful substances, such as microplastic particles, will be precipitated out of bottles that have been used repeatedly for a long time, which will also cause adverse effects on human health. Finally, businesses’ online publicity of bottled water often contains false information, deliberately exaggerating the function of bottled water and fabricating online word-of-mouth evaluations. All these have negative impacts on consumers’ choices and have a bad misleading effect. Therefore, regulating the online market of bottled water, further protecting the environment of the water source, innovating and promoting environmentally friendly packaging, and ensuring the authenticity of online information is of great significance to the establishment of sustainable environmentally friendly consumption. In turn, it will further affect the business online and offline interactive strategies.

However, there is still a lack of quantitative research on risk perception and sustainable bottled water consumption in business online strategies. This study manages to fill the gap in this field. In order to have a deeper understanding of the factors affecting the purchase of bottled water, this study utilized the theory of planned behavior (TPB), which was integrated with risk perception to conduct empirical research, revealing the relationship between risk perception and planned behavior of online bottled water. Therefore, the theoretical contribution of the current study reflects in three sides. First, the theory of perceived risk was extended. This study paper measured the risks of environmental pollution and health hazards from an online bottled water perspective. Second, it enriched the research situation of planned behavior theory. Attitudes, subjective norms, and perceived behavioral control of online bottled water were measured. Third, the theoretical connection between online bottled water perceived risk and planned behavior theory was established, and the specific impact of each variable between the two was revealed, which laid a foundation for further promoting research in this field. At the end of the paper, we discuss managerial guidance for the ecological consumption and sustainable development of online bottled water.

## 2. Literature Review and Theoretical Model

### 2.1. Advantages of Business Online Strategies and Their Environmental Externalities

Business online strategy refers to the dynamic management process to optimize and innovate organizational and operational structure through network technology to construct online business transaction model, so as to better adapt to online consumer demand and thus to guide and create online market. The offline strategies of enterprises focus on price and profit and do not fully consider customers’ needs. Business online strategy focus on the needs and relationships with consumers [16]. It has many advantages in maintaining communication with consumers regarding product R & D, production and sales, allowing customers to actively participate in the enterprise’s product projects, genuinely realizing the value system with customers as the core, and realizing value co-creation and long-term development.

First, reducing the enterprise cost. A business online strategy dramatically reduces the costs invested by enterprises, middlemen and profit dispersion [17]. In the offline strategies of businesses, middlemen always act as a bridge between enterprises and consumers. If products have multiple middlemen, the prices of products will also rise, and these costs will be added to consumers, which damages the interests of consumers and makes it difficult for enterprises to promote products, and seriously affects the competitiveness of products [18]. However, business online strategies can completely avoid such problems. Enterprises reduce the costs of middlemen and physical stores, increase consumer surplus, and enhance product competitiveness [17]. Enterprises will also reduce production risks. In the past, the traditional production mode that was used to determine the production volume was based on experience and speculated consumption dynamics. The online data obtained through an business online strategy can customize production and sales according to the demand of consumers, and the enterprise’s operation risk will be greatly reduced [19].

Second, expanding the market scope. With the help of the Internet, business online strategies can break the constraints of national boundaries, explore regional exchanges, and open up a new global market because the Internet is completely free from time and space constraints [20]. An business online strategy can realize a 24-h business model that no longer limits the store to a corner of a city and expands the business time and scope. It can meet the needs of consumers in different regions and at different purchase times around the clock. Enterprises use the least investment to maximize the degree of synergy and strengthen market control.

Third, enabling enterprises to obtain relatively fair competition opportunities. The online strategy of enterprises provides a development opportunity of fair competition. In the Internet world, there are no restrictions regarding multinational enterprises, state-owned enterprises, central enterprises, and other large enterprises, such that enterprises can have fair access to market information. Therefore, enterprises, especially small- and medium-sized enterprises, can obtain more development opportunities and fair competition opportunities [21].

Fourth, more accurate market positioning. A large amount of customer information support will make the online strategy effective. Receiving and publishing information quickly is the foundation of enterprise survival and development. The Internet enables consumers to query and obtain more personalized information and products, and personalized consumption demand has become a trend [22]. In the offline strategy, enterprises must incur a lot of costs to obtain consumer information. In contrast, the online strategy can greatly weaken the cost problem, allow for the continuous flow of consumer information into enterprises, provide personalized products and services for enterprises, and formulate more accurate positioning for the development of enterprises.

However, while a business online strategy is conducive to the enterprise’s development, it also inevitably produces external environmental problems. The famous economist A. Marshall put forward the externality theory in 1910, and then his student A.C. Pigou enriched and developed this theory. An externality refers to the non-market impact of the activities of producers or consumers on other producers or consumers in actual economic activities [23]. This effect may be beneficial or harmful. Beneficial effects are external economies or positive externalities; harmful effects are called external diseconomies or negative externalities. Environmental pollution and health hazards are external diseconomies that are caused by bottled water production and consumption.

From the perspective of bottled water production externalities, the externality problem is related to the particular attribute of the water source environment itself, that is, the public goods attribute of the water source. As rivers, lakes, groundwater and other water resources are public goods without clear property rights, enterprises and individuals can use water resources and discharge wastes according to their cost-benefit principle, which will inevitably lead to the tendency of abusing water resources and damaging the economic welfare of the surrounding people. The famous thesis “the tragedy of the commons” describes the consequences of the loss of ownership [24]. With the rising demand for bottled water, more enterprises invest in bottled water production, which leads to the over exploitation of water sources. For example, the increase in the number of processing and filling plants in enterprises also produces more sewage discharge, affecting the quality of the water sources. In addition, the massive loss of water source has led to the disorder of the water cycle, dry weather, and a reduction in the number of surrounding animals and plants, which seriously weakens the self- circulation purification function of a water source and causes indirect pollution of the water source. At the same time, after some bottled water is recycled, enterprises do not clean and disinfect the bottle body thoroughly to improve the circulation rate of filling bottles. Therefore, bottled water entering the market again will have an adverse impact on consumers’ health, especially the long-term impact of the enrichment of harmful substances in the body.

From the perspective of bottled water consumption externalities, since Marshall and Pigou put forward the externalities theory in the early 20th century, the internalization of the environmental externality of production behavior through market and administrative means has become an effective way for governments all over the world to solve resource and environmental risks. However, the “direct identity” between production and consumption determined that consumption behavior also has externalities. In fact, with the extensive development of enterprise network strategies, consumers also pay a heavy price in terms of waste, shortage of resources and environmental pollution when they enjoy the convenience, benefits and rich material wealth. Therefore, from the perspective of bottled water, it is also the externality of consumption and indifference to it that aggravates the diffusion of bottled water externalities to a certain extent. On the one hand, consumers’ desire for the convenience and health of bottled water promotes the supply of enterprises, which leads to the negative externalities of production on the environment and human health demonstrated above. On the other hand, the waste generated by consumption, such as non-degradable plastic bottles, brings pollution to the surrounding environment. Under the market mechanism, the increase in processing costs reduces the efficiency of recycling. Therefore, most plastic bottles did not enter the recycling link but are discarded and burned, resulting in secondary pollution to the surrounding environment. In addition, the plastic bottle itself will cause potential long-term harm to the human body due to the addition of chemical components, such as plasticizers and micro-plastics. Consequently, consumers’ consumption behavior must also be considered responsible for the negative externality of bottled water. In other words, without the strong support of a sustainable consumption behavior model, it is impossible to truly realize the sustainable development of enterprises.

### 2.2. Perceived Risk Theory and Online Bottled Water Consumption

Due to the asymmetry of information, consumers cannot accurately predict the results after purchase, and some results may be unpleasant. This kind of “psychological pressure” that produces unpleasant results is the perception of shopping risk. It includes monetary loss, social disapproval, physical damage, product quality problems, and waste of time. Under the influence of risk perception, consumers may change their original attitude towards products, re-evaluate the purchase recommendations given by people around them, and ultimately change their purchase intention and purchase behaviors. Consumers buying bottled water through Internet e-commerce platforms will also be affected by risk perception. This study used risk perception theory and the theory of planned behavior (TPB) to reveal the specific factors involved.

The concept of risk perception originated from psychological research, and later scholars extended this concept to the study of consumer behavior. Professor Raymond Bauer from Harvard University researched the perspectives of the negative impact and uncertainty of risk perception and defined the connotation of risk perception. He pointed out that risk perception is the subjective perception of the objective existence of risks by consumers: the first is consumers’ uncertainty regarding the results of their purchase decisions; the second is the uncertainty of consumers’ satisfaction with the consequences of their purchase decisions [25]. Based on Bauer’s definition of risk perception, many scholars later modified and supplemented the connotation of risk perception.

Specifically, Cox and Rich focused on the various stages of the purchase process and defined risk perception from pre-purchase and post-purchase aspects. It was believed that consumers were uncertain about their decision making before buying and they would have uncertainty about the negative effects of the product on themselves after the purchase [26]. Scholars have continuously enriched and perfected the definition and connotations of risk perception. Cox pointed out that risk perception includes two aspects: financial risk and psychological risk. Jacoby and Kaplan believed that consumers’ risk perceptions included physical risk, financial risk, social risk, functional risk, and psychological risk [27]. In addition, he also empirically studied the impact of these five aspects of risk on overall risk perception. On this basis, other scholars have conducted more in-depth research on the dimensional composition of risk perception. Murray and Schlacter believed that risk perception consists of five aspects, namely, social, financial, performance, psychological, and physical risks [28]. Jarvenpaa and Todd proposed five types of online shopping risk perception: economic, social, functional, personal, and privacy risks [29]. Among them, personal risk is the possibility that individuals will be harmed due to their purchasing behavior. Loss of finances, products, psychological well-being, and time convenience are those risks that are generally recognized by online shoppers [30,31]. In terms of the perceptions and emotions that are induced by an image of a tourist destination, the perception of psychosocial risk and financial risk had a negative impact, while the perception of physical risk had no significant impact on the image of the destination [32]. In the consumer’s use of online shopping application software, the two manifestations of consumer risk perception are the impacts of privacy risk and security risk. Moreover, in cross-cultural contexts, the role of risk perception is different [33].

Summarizing the dimensions of consumer risk perception in online shopping that scholars proposed, this concept mainly includes information, economic, efficacy, time, psychological, privacy, physical, delivery, service, and operational risks. Of course, the risk dimensions that make up the overall risk perception vary depending on the risk context [34]. The dimension of online shopping risk perception is slightly different from the dimension of risk perception in the traditional environment. Consumers have a higher degree of risk perception in the online environment regarding the three aspects of personal finance, time, and product authenticity. With the development of the digital economy model, artificial intelligence and blockchain technology have improved network security. In particular, the security of China’s current major online payment tools (e.g., Alipay and WeChat) has been recognized by consumers, and online consumers have fewer concerns in this field. The gradual popularity of 5G networks has increased network speeds. Chinese netizens are generally satisfied with network speeds, and offline delivery speeds are also very fast. Therefore, consumers’ perception of the risk of time loss is significantly reduced. Consequently, consumers pay more attention to the quality of products in online shopping for online bottled water purchases. Combining the characteristics of bottled water, the risk perception of bottled water in this study was mainly reflected in terms of three aspects: water pollution risk perception (WPRP), non-degradable packaging pollution risk perception (NPPRP), and false information risk perception (FIRP). First, for WPRP, the media often reports the water quality of online bottled water, and consumers are more concerned about bottled water quality. If there is a potential pollution hazard in the source of bottled water, the water quality of its products will inevitably be affected. Second, for NPPRP, many of the materials that are used in plastic bottles are non-degradable, which is also the result of companies out of cost considerations. The production cost of degraded plastics is high, and often few companies are willing to invest in higher costs since it, will affect the company’s profits. Some consumers have realized the importance of environmentally friendly packaging. More and more pollution problems have caused many consumers to realize the harmfulness of plastic bottles to the environment gradually; therefore, they will gradually reduce their consumption of bottled water. Third, for FIRP, many bottled water brands have false information in their Internet promotion, deliberately exaggerating the water efficacy or hiring fake consumers to provide good online reviews to induce consumers to buy their bottled water. The variety and complexity of online information also make it difficult for many consumers to distinguish between genuine and fake information, which constitutes a consumption risk factor.

### 2.3. Theory of Planned Behavior and the Research Framework

In Ajzen’s theory of planned behavior (TPB), three factors affect individual behavior: attitude, subjective norm, and perceptual behavior control [35]. Attitude can be defined as the degree of approval or disapproval of an individual’s evaluation of a behavior. A subjective norm (SN) reflects the individual’s cognition of the social environment, the people’s expectations, and the motivation to adapt to these social environments. Perceptual behavior control (PBC) indicates the ability of an individual to think that they can control the effects of behavior. TPB has shown to be effective in behavioral research in many fields. For example, regarding the environmental behavior of employees in the workplace, the TPB variable explained the three environmental behavior intentions [36]. TPB was used to study the willingness and behavior of farmers to read and use risk information on pesticide labels, where the three main variables of TPB explained farmers’ differences in willingness to read and use labels [37]. The TPB variable explained the willingness to donate blood for higher education students’ voluntary blood donation behavior [38]. Trumbo and O’Keefe [39], Lam [40], and Clark and Finley [41] studied the water conservation behavior intention of community residents in California and China and found that the TPB variables had a strong predictive effect on the intention of green behavior.

**Figure 1:**
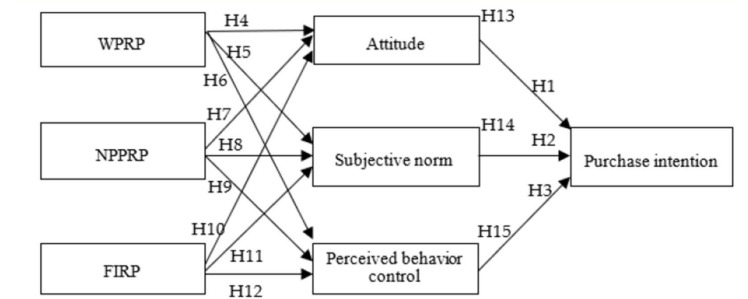
Research framework and hypothesis. Note: WPRP—water pollution risk perception; NPPRP—non-degradable packaging pollution risk perception; FIRP—false information risk perception.

In conclusion, the TPB provides a reliable framework for studying the influencing factors of consumers’ online bottled water purchase intention behavior. The research on the pre-influencing factors of TPB still needs to be combined with other theories to reveal the influencing factors on individual behavior fully. Based on the risk perception theory and TPB theory, this study proposed a model of the influence relationship of purchase intention of bottled water from environmental protection, providing a more comprehensive understanding of the influence mechanism of online bottled water consumption behavior. It also enriches and supplements research on environmentally friendly consumption and sustainable consumption. The specific model is shown in Figure 1 below.

## 3. Research Hypotheses

### 3.1. Purchase Intention of Online Bottled Water Based on TPB

In the early 21st century, with the development of network security, online shopping has gradually won the public’s trust. Moreover, it is accepted by more and more buyers and sellers. For example, goods sold online basically include all types of goods and services, such as books, clothing, food, travel services, hotel services, and electronic products. Compared with physical stores, online platforms offer more goods, convenient purchases, and rapid development. Scholars are also increasingly interested in research in this field. Before 2000, there was only a small amount of research on the subject of online shopping. However, after that, many scholars began to study and investigate online shopping behavior. Consumer online shopping behaviors could help online retailers target potential customers and obtain more benefits by developing and improving online shopping [42].

Research by scholars showed that many factors affect consumers’ online shopping behavior, such as attitudes, subjective norms, perceived behavior control, and risk perception. Ajzen found that the three variables of attitude, subjective norm and perceived behavior control affected the behavioral intention of individuals [35]. The TPB applies to the study of online purchase intention [43]. Consumers’ attitudes, subjective norms and perceived behavior control positively relate to online shopping intention [44].

First of all, “attitude” refers to a person’s positive or negative evaluation of the performance of a particular behavior. Grunert and Ramus also found that factors such as experience, attitude, habits, and ifestyle significantly influence user behavior [45]. Generally speaking, the more affirmative and positive an individual’s attitude towards a particular behavior is, the stronger the individual’s behavior intention will be. As far as this research is concerned, the more positive a consumer’s attitudes toward the safety, health, cleaner, and convenient use of online bottled water are, the stronger their willingness to consume.

Second, a subjective norm (SN) refers to the social pressure an individual feels about undertaking a particular behavior. The research of this study mainly refers to the approval, support, understanding, and recommendation of the consumers regarding the use of online bottled water. The more people around the consumer that approve, support, understand and recommend online bottled water, the stronger the consumer’s willingness to consume.

Finally, the concept of perceptual behavior control (PBC) in the TPB is similar to the connotation of perceptual self-efficacy put forward by social cognition theory. Perceptual behavior control refers to the ability of an individual to control the opportunities and resources needed when taking action. The theory of social cognition claims that personal factors and environmental factors will impact an individual’s behavior, thereby establishing an individual’s self-efficacy, that is, whether an individual believes that they can perform the behavior. The more resources and opportunities they have and the fewer obstacles they anticipate, the stronger their perceptual control over their behavior. According to the theory of planned behavior, consumers’ perceived behavioral control, attitudes, and subjective norms of online bottled water jointly affect purchase intentions (PIs). The stronger an individual’s perceived behavior control over purchasing online bottled water, the higher their willingness to execute this behavior, and the greater the possibility of actual individual actions.

Therefore, this study proposed the following hypotheses:

**Hypothesis 1 (H1)**  *Attitudes regarding online bottled water positively affect the PI for online bottled water*.

**Hypothesis 2 (H2)**  *SNs regarding online bottled water positively affect the PI for online bottled water.*

**Hypothesis 3 (H3)**  *PBC regarding online bottled water positively affects the PI for online bottled water*.

### 3.2. Impact of Risk Perception of Online Bottled Water on Planned Behavior of Consumers

Scholars have analyzed traditional offline consumer behavior [26,27,46]. As the economy develops and technology advances, consumers will face many new risks. Many scholars have also taken into account the new changes in consumers’ risk perception that were brought about by new features, such as virtual networks and technological environments, such that research on the impact of online shopping risk perception is constantly updated. Jarvenpaa and Toddare were the first scholars to study consumers’ risk perception in the B2C field [29]. They referred to the definition from Dowling and Staelin: risk perception is consumers’ subjective perception of uncertainty and negative results during online shopping. Due to the uncertainty of consumers regarding purchasing decisions, risk perception is always present [47].

Liang and Huang found that online consumers’ attitudes and risk perceptions significantly affected consumers’ buying behavior [48]. Online consumers’ trust and risk perception toward online retailers affect their shopping attitude. Liebermann and Stashevsky found a negative correlation between risk perception and consumers’ use of e-commerce shopping [49]. Limayem et al. combined the perception of risk with the perceptions of effectiveness and convenience and analyzed the purchase intention of online consumers [50]. The research results show that convenience, effectiveness, and risk perception all significantly impact online consumers’ purchase intentions, where risk perception is the most significant factor. For this study, as is discussed above, we divided the risk perception of online bottled water into water pollution risk perception (WPRP), non-degradable packaging pollution risk perception (NPPRP), and false information risk perception (FIRP). These risk perceptions will weaken the planned behavior of consumers.

First, when consumers realize that there may be pollution in the water source of bottled water, in the environment around the water source, in the process of water source treatment, or the use of the water source, then the drinking safety, health, and superiority of bottled water compared with tap water will be affected to some extent. From purchasing and drinking online bottled water without concern to the hesitation and exclusion caused by pollution, consumers will have environmental and health anxiety regarding the behavioral consequences of an online drinking water purchase, affecting consumers’ behaviors and attitudes.

In addition, if there is a risk of water pollution, the surrounding people in favor of, support, understand and recommend the use of bottled water will cause doubt and negation. Consumers may think that the surrounding people display encouraging behavior due to not understanding the water pollution; therefore, the consumers will question the encouraging behavior of the surrounding people, and even counter this behavior by trying to correct it, resulting in consumers’ subjective norms being negatively affected to a certain extent.

Finally, the pollution of water sources will shake the concept of consumers’ convenience of online water purchases, and even the previously thought advantages of low time-consumption and low cost will no longer be attractive under the understanding of pollution. In particular, consumers who pay attention to environmental protection and health will take measures to call for improvement or notify the regulatory authorities for punishment. As a result, consumers’ perceived behavior control over the purchase of bottled water will be affected.

Thus, this study proposed the following hypotheses:

**Hypothesis 4 (H4)**  *WPRP of online bottled water negatively affects attitudes regarding online bottled water.*

**Hypothesis 5 (H5)**  *WPRP of online bottled water negatively affects SNs regarding online bottled water*.

**Hypothesis 6 (H6)**  *WPRP of online bottled water negatively affects the PBC regarding online bottled water*.

If consumers realize that the non-degradable packaging of bottled water will cause environmental pollution and harm to the human body, consumers would have concerns and fears about the disposal and negative consequences of the non-degradable packaging after buying bottled water online. The living environment and health are closely related to consumers, and the destruction of the living environment will make consumers feel a sense of crisis. It is inevitable that consumers adjust their behavior by themselves to avoid damage to the environment and health. Thus, the advantages and trust of bottled water (e.g., safety, health, cleanliness, and convenience) are reduced, which affects the behavior and attitude.

Additionally, the pollution caused by the non-degradable packaging of bottled water will cause consumers to doubt and argue when people around them approve of and recommend the use of online bottled water. Furthermore, they will prevent people around them from choosing online bottled water, thus inhibiting the subjective norms of online bottled water.

Lastly, when consumers are aware of the harmfulness of the non-degradable packaging of bottled water, the convenience, time, and money-saving advantages of consumers who choose to purchase bottled water online can be weighed against the dangers. From the perspective of short-term consumption, consumers may use this method to meet their drinking water desire temporarily. However, when considering the sustainability of consumption, consumers may consciously look for drinking water that is convenient, economical, and harmless to the environment and health, or there may be a middle choice, that is, a way that will not have an irreversible impact on the environment and health. In the long run, the consumers’ perceived behavior control will be affected.

Thus, this study proposed the following hypotheses:

**Hypothesis 7 (H7)**  *NPPRP of online bottled water negatively affects attitudes regarding online bottled water*.

**Hypothesis 8 (H8)**  *NPPRP of online bottled water negatively affects SNs regarding online bottled water.*

**Hypothesis 9 (H9)**  *NPPRP of online bottled water negatively affects the PBC regarding online bottled water.*

When consumers find that there may be false publicity in the online bottled water product information, negative information may be hidden in the online bottled water product information, and exaggerated publicity may exist in the function introduction in the online bottled water information, the belief of consumers that the online bottled water has the advantages of safety, health, cleanliness, and convenience will be challenged. As a result, they may believe that the series of health indicators mentioned in the online bottled water sales may be false. This suspicion may subvert all perceptions of online bottled water, trigger a trust crisis, and even issue rights protections and claims, which may have a negative impact on consumers’ attitudes.

Furthermore, the fact that consumers endorse and recommend online bottled water may inspire consumers to want to expose false information about bottled water and think this is a fight against counterfeiting. People who are affected by consumers may give up their positive evaluation of online bottled water and, instead, support consumers in exposing fraudulent practices. Therefore, the FIRP of online bottled water has a negative impact on the subjective norms of online bottled water.

Ultimately, consumers may think that buying online bottled water with false information regarding its convenience and cost advantage may have unpredictable consequences. False information may involve falsifying water quality standards and other parameters, and substandard water quality can greatly harm consumers’ health. Consequently, the seemingly temporary convenience of an online purchase may lead to illness or large medical expenses in the future, which ultimately outweighed the gains, affecting consumers’ PBC.

Thus, this study proposed the following hypotheses:

**Hypothesis 10 (H10)**  *FIRP of online bottled water negatively affects attitudes regarding online bottled water*.

**Hypothesis 11 (H11)**  *FIRP of online bottled water negatively affects SNs regarding online bottled water*.

**Hypothesis 12 (H12)**  *FIRP of online bottled water negatively affects PBC regarding online bottled water*.

### 3.3. Mediation Effect of Consumer Attitudes and Subjective Norms

Based on the above analysis, the risk perception of water source pollution of online bottled water, non-degradable packaging, and online false information negatively impact attitudes and subjective norms. That is, the higher the perceived risk of water pollution, non-degradation packaging, and online false information, the more directly they affect attitudes, subjective norms, and perceived behavior control of consumers regarding online bottled water purchases, thus indirectly affecting the willingness to consume. Therefore, this study puts forward the further hypothesis that attitudes, subjective norms, and perceived behavior control have mediating effects between risk perception of online bottled water and the purchase intention of bottled water:

**Hypothesis 13 (H13)**  *Attitudes regarding online bottled water have a mediating effect in the influence of WPRP (a), NPPRP (b), and FIRP (c) on the purchase intention for bottled water*.

**Hypothesis 14 (H14)**  *SNs regarding online bottled water have a mediating effect in the influence of WPRP (a), NPPRP (b), and FIRP (c) on the purchase intention for bottled water*.

**Hypothesis 15 (H15)**  *The PBC regarding online bottled water has a mediating effect in the influence WPRP (a), NPPRP (b), and FIRP (c) on the purchase intention for bottled water*.

## 4. Methodology

### 4.1. Measures

This research was divided into two parts, pre-survey and formal survey. The pre-survey was conducted to verify the reliability and validity of the scale, while a formal survey was conducted to test the hypotheses. In the pre-survey, a questionnaire was built and samples were collected through the network survey platform WJX.cn to enable the interviewees to reflect their true feelings about the perceived risk of online bottled water and their planned behaviors. The measures of the related constructs were adapted from previous studies according to the contexts of online bottled water consumption to form the pre-survey scales and were tested through the method of reliability and validity by using the statistical tools in SPSS and AMOS. In the formal survey, we used the questionnaire tested in the pre-survey through the network survey platform WJX.cn to carry on the random sampling. Then, the reliability and validity of the sample were tested using AMOS, in order to verify the hypotheses proposed in this study. The measurement of perceived risk involves three constructs, each of which includes three items adapted from Jacoby and Kaplan [27] (see Table A1). At the same time, consumers’ planned behavior was adapted from Ajzen [35] (see Table A2), including four constructs: behavior, subjective norms, perceived behavior control and purchase intention. In order to make each item better reflect the online consumption of bottled water, we interviewed thirty consumers to confirm the rationality of the theoretical framework of this study and the specific content of the scale. We also invited five experts and scholars to discuss the questionnaire and reached a consensus, thus ensuring the content validity of the questionnaire. Each item was measured with a five-level Likert scale, where 1 denoted “strongly disagree”, and 5 denoted “strongly agree”.

### 4.2. Data Collection

The study objects of this study were mainly consumers who bought bottled water online; therefore, the questionnaire is mainly made and released through the WJX.cn. In order to ensure the validity and integrity of the data, the author inquired about the Baidu Index and the related analysis reports of bottled water in advance, took the main online bottled water consumers as the research objects, and cooperated with WJX.cn platform to collect questionnaires extensively and directionally. In order to make the sample more representative and reduce the deviation, in the formal survey, we investigated six major cities in China distributed in different directions, namely, Beijing (17.2%), Xi’an (15.2%), Shanghai (17.4%), Wuhan (15.7%), Chengdu (15.6%), and Guangzhou (18.9%). The respondents first needed to answer a filter question during each investigation process: Do you often buy bottled water online? The questionnaire ended if the answer was no; otherwise, they continued to answer the follow-up questions. The following item is a guide: “imagine the situation when you buy bottled water on the Internet. You perceive that bottled water produces certain risks, such as water source pollution, non-degradable packaging pollution and false network information.” Then, participants were asked to answer questions about the perceived risk of bottled water and planned behavior. The questionnaire ended with the demographic questions, and the participants received 2 RMB WeChat red envelopes as a token of appreciation for their participation. 

## 5. Data Analysis and Results

### 5.1. Pre-Survey Analysis

#### 5.1.1. Reliability Test

With the help of WJX.cn (online questionnaire platform), 97 samples were collected online. In order to further ensure the quality of the data, all the samples with the same answer and the samples with an answer time of less than 60 seconds were eliminated; a total of 80 valid questionnaires were identified. The effective recovery rate of the questionnaire was 82.5%. Then we carried out the reliability and validity tests of the pre-survey data.

Reliability analysis is mainly done to test the internal consistency of each item in the questionnaire. This study judged the internal consistency of each item by testing the reliability of the questionnaire (selecting the correlation between the revised item and the total score and the Cronbach’s alpha after deleting the item). The reliability test results (Table A1) showed that the reliability of the risk perception scale of online bottled water was 0.939, while the risk perception scale of water pollution, risk perception scale of non-degradable packaging pollution, and risk perception scale of online false information were 0.900, 0.896, and 0.924 respectively, which indicated that the internal consistency of each dimension scale met the requirement, that is, the reliability of each dimension scale was fairly good [51].

At the same time, this was further verified using the correlation between the revised item and the total score and the Cronbach’s alpha after deleting the item. Table A1 shows that the correlation between the item and the total score after each item was revised was greater than 0.7, and the Cronbach’s alpha after the item was deleted was less than the Cronbach’s alpha of the dimension scale when it was not deleted, which further showed that the reliability of the risk perception scale of online bottled water consumer was fairly good.

The reliability test results (Table A2) of planned behavior for the online bottled water showed that the Cronbach’s alpha of the attitude, subjective norm, perceived behavior control, and behavior intention dimensions, were 0.900, 0.912, 0.901, and 0.919, respectively, and 0.944 for the total scale, which indicated that the reliability of the scale met the requirement. At the same time, Table A2 showed that the correlations between the revised items and total score were all greater than 0.7, which meant that the reliability of the planned behavior scale was fairly good.

#### 5.1.2. Validity Test

The pre-survey validity analysis of this study mainly tested the structural validity of the scale questionnaire and evaluated the validity of the scale’s measurement results, mainly through exploratory factor analysis (EFA). The analysis results showed that KMO result of the risk perception scale was 0.916, which is greater than 0.9; the approximate chi-square value was 678.388; and *p* value was 0.000, which is much less than 0.01, reaching a significant level, indicating that the structural validity of the scale was fairly ideal [52]. In addition, the KMO result of the behavioral planning scale was 0.906, which is greater than 0.9; the approximate chi-square value was 958.750; and *p* value was 0.000, which is much less than 0.01, reaching a significant level, indicating that the structural validity of the scale was relatively ideal.

Principal component analysis (PCA) was used to conduct factor analysis on the online bottled water perceived risk scale and the planned behavior scale, with the rotation axis method of the maximum variance method. The results (Table A3 and Table A4) indicated that the factor number of each scale was consistent with each concept dimension proposed in this study, the factor loading of each item was greater than 0.45; the commonness of each item was greater than 0.02; and the cumulative explained variance was greater than 50%. Furthermore, there was no one factor that explained most of the data variance, which indicated that the risk of common method deviation in this study did not pose a threat.

### 5.2. Statistical Data Analysis

#### 5.2.1. Sample

The measurements of constructs in this study were showed to have reliability and validity in the pre-survey. The process of the formal survey was the same as that of pre-investigation. In the formal survey, 459 questionnaires were collected through an online survey platform (i.e., WJX.cn) using the scales that we tested by the pre-survey. At the same time, in order to further ensure the quality of data, all the samples with the same answer and the samples with an answer time of less than 60 seconds were eliminated. Finally, 401 valid questionnaires were obtained, where the questionnaire recovery rate was about 87.36% (Table 1).

According to the distribution of gender, age, occupation, and educational background of the sample, it basically conformed to the profile of the main consumers of online bottled water.

#### 5.2.2. Reliability Test

The reliability test results (Table 2) showed that the reliability of the risk perception scale of online bottled water was 0.931, while the risk perception scale of water pollution, risk perception scale of non-degradable packaging pollution and risk perception scale of online false information were 0.891, 0.907 and 0.892, respectively, which indicated that the internal consistency of each dimension scale meets the requirement, that is, the reliability of each dimension scale was fairly good. In addition, the reliability test of planned behavior regarding online bottled water showed that the Cronbach’s alpha of attitude, subjective norm, perceived behavior control, and behavior intention dimensions were 0.908, 0.911, 0.875, and 0.912, respectively, and 0.937 for the total scale, which indicated that the reliabilities of the scales meet the requirement. At the same time, Table 2 shows that the correlations between the revised items and the total score were all greater than 0.7, which meant the reliability of the planned behavior scale was relatively good. The Cronbach’s alpha after the item was deleted was less than the Cronbach’s alpha of the dimension scale when it was not deleted, further showing that the scale reliability of online bottled water is fairly good. In summary, the reliabilities of the overall questionnaire and the dimensional scale were reasonably good.

#### 5.2.3. Validity Test

The validity of the formal survey was tested using the confirmatory factor analysis (CFA) model with AMOS, including convergent validity and discriminant validity. The model fit indices of CFA (χ²/df = 1.442, GFI = 0.944, AGFI = 0.929, CFI = 0.995, RMSEA = 0.019, SRMR = 0.0220) met the requirements suggested by Marsh et al. [53] and Bentler and Bonett [54]. Convergent validity identifies whether the measurement item should be in the same factor, while discriminant validity identifies whether the measurement item should not be in the same factor [55].

First, Fornell and Larker pointed out in the study on the convergent validity test that an average variance extracted (AVE) greater than 0.50 is an ideal value [56]. The results of this study through CFA are shown in Table 3. The AVE of each dimension scale was 0.672, 0.709, 0.735, 0.712, 0.718, 0.706, and 0.723, which were all greater than 0.5. The standard loading coefficient of each item was greater than 0.8, and the composite reliability (CR) was greater than 0.6, indicating that the convergent validity of the questionnaire was relatively good.

Second, regarding the test of discriminant validity, Fornell and Larcker pointed out that if the square root of a latent variable’s AVE is greater than the value of the corresponding coefficient, it indicates that the discriminant validity between variables is ideal [56]. In this study (Table 4), the square roots of AVE for each variable were 0.820, 0.842, 0.857, 0.844, 0.848, 0.840, and 0.850, which were all greater than the correlation coefficient between the variables. This showed that the questionnaire in this study had ideal discriminant validity.

Therefore, based on the CFA analysis, the validity of the questionnaire in this study was relatively good, and the scale could accurately measure the variables that needed to be measured. Thus, the study could proceed to the next step, which was hypothesis testing.

#### 5.2.4. Hypothesis Testing

A structural equation model (SEM) is a multivariate statistical technique that combines factor analysis and path analysis. It is a relatively good method in social science research regarding latent variables. Structural equation modeling can consider and process multiple dependent variables at the same time. It can allow independent variables and dependent variables to contain measurement errors, and at the same time, can estimate the factor structure and factor relationship, as well as the goodness of fit of the entire model.

First, we tested the goodness of fit, which is the most important indicator of the quality of the SEM. The goodness of fit index is the degree of consistency between the hypothetical theoretical model and the actual data. The higher the model fit, the higher the degree of congruence between the theoretical model and the actual data. In this study, all indicators meet the standard of goodness of fit (χ²/df = 1.339 < 3, GFI = 0.934 > 0.9, AGFI = 0.918 > 0.9, CFI = 0.988 > 0.9, RMSEA = 0.029 < 0.05, SRMR = 0.0383 < 0.05), indicating that hypothesis testing was able to be performed [57,58,59].

Furthermore, the test results of the significance of the path relationship of the model variables are shown in Table 5. The R2 value showed that the research model explained 54.5% of the variance for PI, indicating that the theory of planned behavior had a fair explanatory power in terms of explaining the consumer’s behavior toward bottled water. Additionally, compared with the consumer planned behavior model without the external influence of risk perception (50.9% of the variance for PI), the model’s explanatory power was improved by 3.6%. In addition, The R2 value also showed that the risk perception factors toward bottled water could better explain the influencing factors toward planned behavior toward bottled water. The model explained 66.6% of the variance for AT, 58.3% of the variance for SN and 65.4% of the variance for PBC, respectively. In terms of the significance of the path coefficient (PC) of the relationship between variables (Table 5), AT had a significant effect on PI (PC = 0.226, C.R. = 3.586, *p* < 0.001), and SN had a significant effect on PI (PC = 0.342, C.R. = 5.643, *p* < 0.001). The effect of PBC on PI was significant (PC = 0.318, C.R. = 4.707, *p* < 0.001), thus H1, H2, and H3 were supported. WPRP had a significant negative effect on AT (PC = −1.024, C.R. = −8.527, *p* < 0.001), SN (PC = −0.717, C.R. = −6.241, *p* < 0.001) and PBC (PC = −0.843, C.R. = −7.377, *p* < 0.001), thus H4, H5, and H6 were supported. However, NPPRP did not significantly affect AT, SN, and PBC, showing that H7, H8, and H9 were not verified. FIRP had a significant effect on SN (PC = −0.142, C.R. = −2.152, *p* < 0.001), rather than on AT (*p* = 0.202) and PBC (*p* = 0.076), indicating H11 was supported while H10 and H12 were not.

Ultimately, we tested the mediating effect of the latent variables AT, AN and PBC by constructing nine mediating models using AMOS. The bootstrap method can better reveal the mediating effect [60]. Therefore, the bootstrap method was used in AMOS to test the model, where the number of executions is 5000, and the bias-corrected confidence interval was set to 95%. The results showed that the fitting degree of each model met the analysis requirements χ/ df < 3, GFI > 0.9, AGF > 0.9, CFI > 0.9, RMSEA < 0.05, SRMR < 0.05). The specific model fit is shown in Table 6.

Further mediating effect indicators showed that (as shown in Table 7), AT (95% CI = [−0.052 ~ 0.263]), SN (95% CI = [−0.215 ~ 0.085]), and PBC (95% CI = [−0.117 ~ 0.208]) did not play mediating roles in the impact of WPRP on PI because the 95% bootstrap confidence interval included 0, therefore, H13a, H13b, and H13c were not supported. However, AT (95% CI = [−0.284 ~ −0.130]), SN (95% CI = [−0.315 ~ −0.163]), and PBC (95% CI = [−0.299 ~ −0.139]) had partial mediating effects on PI because the 95% bootstrap confidence interval did not include 0, and the direct effect of NPPRP on PI (DE model 4 = −0.484, DE model 5 = −0.455, DE Model 6 = −0.475) was significant; thus H14a, H14b, and H14c were verified. Similarly, AT (95% CI = [−0.336–0.174]), SN (95% CI = [−0.336–0.174]), and PBC (95% CI = [−0.325 ~ −0.140]) played partial mediating roles in the effect of FIRP on PI because the 95% bootstrap confidence intervals did not include 0, and the direct effect of FIRP on PI (DE model 7 = −0.387, DE model 8 = −0.387, DE model 9 = −0.408) was significant. Therefore, H15a, H15b, and H15c were verified.

## 6. Discussion of the Results

Based on the theory of risk perception and planned behavior, this study empirically explored the impacts of three dimensions of risk perception of online bottled water on AT, SN, PBC, and the mediation effects. We constructed a structural equation model to test the hypotheses, which indicated the important influences of the perceived risk dimensions. Such perception is of significance, as consumer perceived risk is detrimental to purchase intention, particularly when the water source environment is severely deteriorating severely and the awareness of health and environment protection is becoming stronger than ever before [15].

First, it was evident that the three dimensions of planned behavior, i.e., AT, SN, and PBC, had significant positive effects on online bottled water purchase intention. This confirmed previous research showing that the TPB model effectively predicts human behavior in certain contexts [36,37,38]. It is worth noting that, compared with AT and PBC, SN had the greatest impact on purchase intention, indicating that people around consumers played an essential role in consumers’ purchase intention of online bottled water, which demonstrated that consumers paid more attention to the opinions of those around them about online bottled water, compared with AT and PBC. The second important factor was PBC, and the last one was AT. The development of the Internet has facilitated the channels for consumers to buy bottled water. Consumers can buy bottled water at any time through online shopping platforms, which are not limited by the time and place of purchase. The price of bottled water is relatively low, which enhances the perceived behavior control of consumers to buy bottled water. Additionally, consumers have gradually recognized that bottled water is safer and easier to use than tap water, forming a positive attitude towards bottled water consumption, thus improving consumers’ purchase intention.

Second, the risk perceptions of online bottled water, i.e., WPRP, NPPRP, and FIRP, had different impacts on AT, SN and PBC [39]. Specifically, WPRP had significant adverse effects on AT, SN, and PBC, affecting AT the most. The reason was that bottled water quality determines consumers’ understanding of bottled water safety and health, and it is also the core requirement of bottled water. Once the water source cannot be guaranteed, consumers will reduce their subjective attitude toward bottled water. From another point of view, it was also explained that bottled water brands promoting water resources quality can often arouse the attention of consumers, stimulate their desire to buy, and achieve good market performance. For example, China’s Nongfu Spring brand emphasizes that “we do not produce water, we are the porters of nature” and “Nongfu Spring is a little sweet” in brand building to establish a natural, healthy and safe brand association to consumers and become the leading brand of bottled water in China. Furthermore, the negative impact of WPRP on PBC and SN, whose impact was lower than that of AT, suggested that the contamination of water sources reduced the support of people around consumers for bottled water, and consumers’ perceptions of the convenience, cost, and time economy of bottled water are reduced. In addition, FIRP had a negative impact on SN. However, FIRP had no significant negative impact on AT and PBC, indicating that exaggerated publicity and false information about bottled water on the Internet mainly reduced the influence of people around on consumers, rather than the influence of AT and PBC. For some false information on the Internet, consumers are mainly influenced by the people around them; that is to say, they form the judgment of false information from interpersonal communication. This shows that many online sales of bottled water pay special attention to the effect of word of mouth. Once negative word of mouth is formed, it will directly inhibit the role of people around consumers in recommending the brand. Finally, NPPRP had no significant negative effect on AT, SN and PBC, indicating that consumers did not pay attention to the pollution caused by non-degradable bottled water packaging and were not aware of the pollution caused by the packaging of bottled water to the surrounding environment. Moreover, the environmental problems that are associated with bottled water packaging did not make people less supportive of and less likely to recommend bottled water. This also explains the subjective source of environmental pollution caused by bottled water at present. Consumers all proceed from their interests in order to satisfy their own consumption needs and ignore the negative effects of consumption on the environment. Therefore, the environmental awareness of bottled water consumption needs to improve.

Third, AT, SN, and PBC partially mediated the influence of NPPRP on purchase intention. The above research results showed that the direct effects of NPPRP on AT, SN, and PBC were not significant, while the negative indirect effects of NPPRP on PI were through each of AT, SN, and PBC, indicating the importance of AT, SN, and PBC in the impact of NPPRP on PI and the importance of the overall analysis of the model. We could not ignore the overall effect just because the parts were not significant. Among the negative indirect effects of NPPRP on PI, SN accounted for the largest proportion (33.96%) of the total effect, indicating that consumers’ perception of pollution risk caused by non-degradable packaging of bottled water mainly reduced consumers’ willingness to buy bottled water through SN. The people around them could influence consumers to buy bottled water, but when the risk of contamination from bottled water packaging was realized, it could inhibit consumers’ purchase intention.

Fourth, the negative indirect effect of AT between FIRP and PI was the same as that of SN (39.25%), while the indirect negative effect of PBC was the least, illustrating that the negative effect of FIRP on PI was mainly through AT and SN. In combination with the above results, AT and PBC were not significantly affected by FIRP but played a significant negative and indirect role in FIRP’s influence on PI, which also demonstrated the necessity of analyzing the overall relationship of the model where the roles of AT and PBC in the overall theoretical model could not be ignored. False and exaggerated information about bottled water on the Internet could change consumers’ attitudes toward bottled water. They did not blindly follow the people’s recommendation information, thus reducing their perceived behavioral control over bottled water and ultimately weakening the consumers’ willingness to buy it.

Finally, AT, SN, and PBC had no significant indirect effects on the negative effects of WPRP on PI, but they all showed significant negative direct effects. This also showed that the quality of bottled water has a substantial direct effect on consumer purchase. The quality of water is the core appeal of bottled water. If it could not be guaranteed, consumers’ attitudes toward bottled water directly changed, where their acceptance of the recommendation intention of the people around them, their perceived behavioral control over bottled water and their purchase intention were all reduced. Therefore, special attention to WPRP is required. As suggested by Ajzen, this result demonstrates that the TPB model would show different influence path relationships and need to be combined with specific problems to disclose the particular connotation under different contexts.

## 7. Conclusions

### 7.1. Theoretical Contributions

This study extended the current understanding of consumers’ risk perception regarding purchase intention, which has gained momentum in recent years [33]. The era of the Internet information explosion has, on the one hand, accelerated the decision-making process of consumers, but on the other hand, it also increases the cost of decision-making. When people perceive risks or benefits, they will make corresponding trade-offs and decisions [26,34].

To start with, this study puts forward the risk perception dimensions of online bottled water based on environmental responsibility, including water pollution risk perception (WPRP), non-degradable packaging pollution risk perception (NPPRP), and false information risk perception (FIRP), and expanded the dimension connotation of risk perception in online shopping from the qualitative aspect [25,26,34]. Through empirical research, this study verified the influence path of each dimension of online bottled water risk perception on attitudes (ATs), subjective norms (SNs) and perceived behavioral control (PBC) and supplemented the current literature on the relationship between perceived risk and behavioral intention. This paper explained the role of different perceived risk dimensions in the online bottled water consumption situation, expanded the understanding of the role of risk perception quantitatively, and provided ideas for the measurement of shopping risk perception in the online shopping situation [27].

Furthermore, echoing the Theory of Planned Behavior, this study examines the purchase intention of online bottled water consumers from risk perception. Previous studies showed the validity of the theory of planned behavior, and the necessity of testing it differently [36,37]. This study verified the relationship between risk perception and planned behavior by constructing the structural equation model, expanding the theory of planned behavior, and understanding the relationship between risk perception and planned behavior theory [34].

Ultimately, this study showed that consumers’ perceived risk of online bottled water affected their purchase intention through the mediating roles of AT, SN, and PBC, which have different indirect effects. This study developed our understanding of the online purchase mechanism under risk perception, especially in terms of how it affects consumers’ online bottled water purchase intentions.

### 7.2. Managerial Implications

The essence of the external diseconomy of environmental pollution and health hazards that are caused by online bottled water is the socialization of private costs. Therefore, to fundamentally solve this problem, it is necessary to internalize the external costs, to let the polluters’ pollution drawbacks affect their production or consumption decisions and avoid the spillovers of costs [61].

First, the source of water and the surrounding environment are related to the quality of bottled water. Online purchases will cause consumers to perceive the risk of pollution of bottled water sources, directly affecting consumers’ AT, SN, PBC, and PI, which are influenced most adversely for online bottled water. Bottled water businesses should not regard online purchases as a convenient way to conceal the pollution of water sources, which will cause environmental problems and consumer health problems and produce irreparable adverse effects. After realizing this direct influence mechanism, Bottled water businesses should regard online purchasing as an important opportunity to turn the risks that consumers are aware of into opportunities. Bottled water businesses should identify potential environmental hazards, optimize the construction of water sources, improve the environment for bottled water production and processing, and supervise and consolidate the ecological construction of the surrounding environment of the water sources to reduce the risk of water source pollution. The government should also carry out strong supervision on the environmental pollution of water sources. For example, strict examinations and approval processes should be carried when constructing factories near a water source, and strict production and pollution detection standards should be established to realize the sustainable development of the water source. In short, the direct negative influence of WPRP on AT, SN, PBC, and PI should be paid much more attention by online bottled water businesses, as well as governments.

Second, the online shopping process has a different supply chain from offline shopping, and its simplified process makes it easier for bottled water businesses to abuse non-degradable packaging, where some irregularities occurred in the process of recycling, processing, and utilizing packaging. This will enhance consumers’ NPPRP and reduce PI through the indirect effects of AT, SN, and PBC. Bottled water businesses should not regard online purchases as a hotbed for abusing non-degradable packaging and avoiding responsibility for protecting the environment. The increased pollution risk of non-degradable packaging will have a profound impact on the environment and human health. The act of focusing only on short-term interests and ignoring long-term interests will bring irreparable losses. With the improvement of consumers’ risk perception, their purchase intention will change. Therefore, bottled water businesses should regard online channels to disseminate environmental awareness and behavior and strive to enhance social influence. Bottled water businesses should work hard to develop and optimize environmentally friendly packaging for bottled water, standardize the recycling, treatment and utilization process of non-degradable packaging, and actively advocate purchasing bottled water with environment-friendly packaging. This can reduce the pollution risk of non-degradable packaging, thereby improving consumer attitudes, subjective norms and perceived behavior control, ultimately promoting consumers’ purchase intentions.

Third, consumers do not have sufficient professional background knowledge, and the amount of information on the Internet is enormous. Consumers purchase bottled water online, which increases the difficulty of information supervision and identifying false information. Bottled water businesses can reduce the FIRP of online bottled water to influence AT, SN, and PBC, consequently affecting consumers’ PI. Bottled water businesses should not regard online purchasing platforms as a favorable place for disseminating false information. On the one hand, excessive publicity or false publicity will seriously mislead society and consumers. On the other hand, insufficient supervision and identification of false information on the Internet will condone the continuous occurrence of water source pollution and non-degradable packaging pollution. If unscrupulous bottled water businesses use these, they will have long-term adverse effects on the environment and consumer health. As a result, consumers’ risk perception is stimulated, and it is more difficult to change. Therefore, businesses should actively cooperate with the government and online platforms’ water source and packaging identification specifications to obtain the corresponding identification certificates and identification marks. In addition, businesses can introduce appropriate online information monitoring measures, such as allowing consumers to learn about offline water sources and processing plants through online platforms. In short, bottled water businesses should actively cooperate with government agencies and online platforms for authentic information verification to advance the development of sustainable consumption.

Overall, although it is difficult to determine the compensation price of water resources property rights, businesses, governments and the public should play decisive roles in limiting the external diseconomy behavior. Therefore, the intervention of government regulatory agencies, the restriction of policies, the enforcement of laws, the popularization of environmental education and the promotion of business and public ecological awareness are of great significance to resolve the pollution risk of bottled water, promote the optimization of enterprises’ online strategies, and achieve sustainable consumption.

### 7.3. Limitations and Further Research

There are some limitations and future research avenues that are worth considering regarding this study. First of all, from the perspective of environmental protection, the risk perception factors of online bottled water consumers include more than the three main factors mentioned in this paper. Future research can continue to explore a wider range of factors to expand the connotation of risk perception, as was suggested by Conchar [34], to more comprehensively reveal the impact of risk perception on consumers’ purchase intention.

Second, the research object of this study was mainly Chinese consumers. Although there are vast quantities of online consumers in China, we can also conduct research in other countries to verify the cross-cultural nature of the model. Chopdar showed that in the cross-cultural context, the role of perceived risk is different [33]. Therefore, the difference in mechanism regarding the impact of risk perception on planned behavior between different countries need exposing in future studies.

Third, our research identifies the mediating mechanism of risk perception on purchase intention. However, consumers with different personalities and purchase motives may have different mediating effects, and this mediating mechanism is also worth considering.

## Figures and Tables

**Table 1:** Distribution of sample demographic variables.

		Frequency	Percentage
Gender	Male	209	52.1
	Female	192	47.9
Age (years)	Under 20	44	11.0
	20–30	145	36.2
	30–40	150	37.4
	Over 40	62	15.5
Occupation	Company employee	160	39.9
	Civil servant	115	28.7
	Student	114	28.4
	Freelancer	10	2.5
	Other	2	0.5
Educational background	Junior high school and below	44	11.0
	Senior high school or technical secondary school	117	29.2
	Junior college	120	29.9
	Bachelor’s degree and above	120	29.9

**Table 2:** Reliability test.

Construct	Item	Correlation ^a^	Cronbach’s Alpha ^a^	Cronbach’s Alpha
WPRP	WPRP1	0.752	0.863	0.891
	WPRP2	0.749	0.864	
	WPRP3	0.755	0.861	
	WPRP4	0.783	0.851	
NPPRP	PCRP1	0.776	0.884	0.907
	PCRP2	0.793	0.878	
	PCRP3	0.785	0.881	
	PCRP4	0.802	0.874	
FIRP	FIRP1	0.796	0.841	0.892
	FIRP2	0.780	0.855	
	FIRP3	0.791	0.845	
AT	BA1	0.790	0.882	0.908
	BA2	0.780	0.885	
	BA3	0.793	0.881	
	BA4	0.805	0.876	
SN	SN1	0.794	0.885	0.911
	SN2	0.816	0.878	
	SN3	0.793	0.886	
	SN4	0.786	0.888	
PBC	PBC1	0.752	0.830	0.875
	PBC2	0.768	0.816	
	PBC3	0.760	0.824	
PI	PI1	0.817	0.881	0.912
	PI2	0.800	0.886	
	PI3	0.791	0.890	
	PI4	0.793	0.889	

Note: WPRP—water pollution risk perception; NPPRP—non-degradable packaging package pollution risk perception; FIRP—false information risk perception; AT—attitude; SN—subjective norm; PBC—perceived behavior control; PI—purchase intention; Correlation ^a^—correlation between the revised item and the total score; Cronbach’s alpha ^a^—Cronbach’s alpha after deleting the item.

**Table 3:** Convergent validity.

Item		Construct	Estimate	S.E.	C.R.	Standardized Estimate	CR	AVE
WPRP 4	←	WPRP	1.000			0.835	0.891	0.672
WPRP 3	←	WPRP	0.942 ***	0.048	19.654	0.813		
WPRP 2	←	WPRP	0.952 ***	0.048	19.821	0.817		
WPRP 1	←	WPRP	0.939 ***	0.048	19.681	0.813		
NPPRP 4	←	NPPRP	1.000			0.850	0.907	0.709
NPPRP 3	←	NPPRP	0.953 ***	0.046	20.627	0.838		
NPPRP 2	←	NPPRP	0.985 ***	0.047	21.034	0.848		
NPPRP 1	←	NPPRP	0.978 ***	0.048	20.350	0.831		
FIRP3	←	FIRP	1.000			0.852	0.893	0.735
FIRP2	←	FIRP	1.007 ***	0.049	20.739	0.853		
FIRP1	←	FIRP	1.021 ***	0.048	21.190	0.867		
AT4	←	AT	1.000			0.865	0.908	0.712
AT3	←	AT	0.960 ***	0.045	21.421	0.842		
AT2	←	AT	0.942 ***	0.045	20.895	0.829		
AT1	←	AT	0.959 ***	0.045	21.289	0.839		
SN4	←	SN	1.000			0.832	0.911	0.718
SN3	←	SN	1.019 ***	0.051	20.145	0.840		
SN2	←	SN	1.098 ***	0.051	21.409	0.875		
SN1	←	SN	1.044 ***	0.052	20.248	0.842		
PBC3	←	PBC	1.000			0.826	0.878	0.706
PBC2	←	PBC	0.981 ***	0.051	19.214	0.846		
PBC1	←	PBC	0.980 ***	0.052	18.984	0.838		
PI4	←	PI	1.000			0.843	0.913	0.723
PI3	←	PI	1.029 ***	0.050	20.681	0.841		
PI2	←	PI	1.018 ***	0.048	21.014	0.850		
PI1	←	PI	1.046 ***	0.048	21.688	0.867		

Note: *** *p* < 0.001; WPRP—water pollution risk perception; NPPRP—non-degradable packaging package pollution risk perception; FIRP—false information risk perception; AT—attitude; SN—subjective norm; PBC—perceived behavior control; PI—purchase intention; CR—composite reliability; AVE—average variance extracted; S.E.—standard error; C.R.—critical ratio for the difference.

**Table 4:** Discrimination validity.

	WPRP	NPPRP	FIRP	AT	SN	PBC	PI
WPRP	0.820						
NPPRP	0.787 ***	0.842					
FIRP	0.751 ***	0.576 ***	0.857				
AT	−0.806 ***	−0.630 ***	−0.570 ***	0.844			
SN	−0.746 ***	−0.598 ***	−0.627 ***	0.631 ***	0.848		
PBC	−0.794 ***	−0.599 ***	−0.649 ***	0.646 ***	0.601 ***	0.840 ***	
PI	−0.791 ***	−0.659 ***	−0.631 ***	0.602 ***	0.634 ***	0.612 ***	0.850

Note: *** *p* < 0.001; the diagonally bolded numbers are square roots of AVE; non-diagonal numbers are latent variable correlations; WPRP—water pollution risk perception; NPPRP—non-degradable packaging package pollution risk perception; FIRP—false information risk perception; AT—attitude; SN—subjective norm; PBC—perceived behavior control; PI—purchase intention.

**Table 5:** Results of structural equation model.

Hypothesis	PC	S.E.	C.R.	Hypothesis Supported?
H1: AT→PI	0.226 ***	0.063	3.586	Yes
H2: SN→PI	0.342 ***	0.061	5.643	Yes
H3: PBC→PI	0.318 ***	0.068	4.707	Yes
H4: WPRP→AT	−1.024 ***	0.120	−8.527	Yes
H5: WPRP→SN	−0.717 ***	0.115	−6.241	Yes
H6: WPRP→PBC	−0.843 ***	0.114	−7.377	Yes
H7: NPPRP→AT	0.016	0.073	0.224	No
H8: NPPRP→SN	−0.035	0.074	−0.472	No
H9: NPPRP→PBC	0.049	0.072	0.676	No
H10: FIRP→AT	0.083	0.065	1.277	No
H11: FIRP→SN	−0.142 *	0.066	−2.152	Yes
H12: FIRP→PBC	−0.114	0.064	−1.774	No

Note: *** *p* < 0.001; * *p* < 0.05; WPRP—water pollution risk perception; NPPRP—non-degradable packaging package pollution risk perception; FIRP—false information risk perception; AT—attitude; SN—subjective norm; PBC—perceived behavior control; PI—purchase intention; PC—path coefficient; S.E.—standard error; C.R.—critical ratios for difference.

**Table 6:** Model fit indices.

Model	χ^2^	df	χ^2^/df	GFI	AGFI	CFI	RMSEA	SRMR
Model 1: WPRP→AT→PI	42.628	51	0.836	0.983	0.973	1.000	0.000	0.0149
Model 2: WPRP→SN→PI	63.187	51	1.239	0.974	0.960	0.967	0.024	0.0254
Model 3: WPRP→PBC→PI	58.268	41	1.421	0.974	0.959	0.995	0.032	0.0217
Model 4: NPPRP→AT→PI	52.969	51	1.039	0.979	0.968	0.999	0.010	0.0190
Model 5: NPPRP→SN→PI	51.045	51	1.001	0.980	0.970	1.000	0.001	0.0190
Model 6: NPPRP→PBC→PI	49.244	41	1.201	0.978	0.965	0.997	0.022	0.0204
Model 7: FIRP→AT→PI	46.265	41	1.1280	0.979	0.967	0.998	0.018	0.0151
Model 8: FIRP→SN→PI	47.362	41	1.155	0.979	0.966	0.998	0.020	0.0193
Model 9: FIRP→PBC→PI	51.184	32	1.599	0.975	0.957	0.993	0.039	0.0211

**Table 7:** Test results of the mediation effects.

Model	Hypothesis	TE	DE	IE	ER	95% CI	Mediation?	Hypothesis Supported?
Model 1: WPRP→AT→PI	H13a	−0.937 ***	−1.033 ***	0.096	-	[−0.052 ~ 0.263]	No	No
Model 2: WPRP→SN→PI	H13b	−0.947 **	−0.871 **	−0.076	-	[−0.215 ~ 0.085]	No	No
Model 3: WPRP→PBC→PI	H13c	−0. 934 **	−0.972 **	0.038	-	[−0.117 ~ 0.208]	No	No
Model 4: NPPRP→AT→PI	H14a	−0.686 ***	−0.484 ***	−0.202 ***	29.45%	[−0.284 ~ −0.130]	Partial	Yes
Model 5: NPPRP→SN→PI	H14b	−0.689 ***	−0.455 ***	−0.234 ***	33.96%	[−0.315 ~ −0.163]	Partial	Yes
Model 6: NPPRP→PBC→PI	H14c	−0.688 ***	−0.475 ***	−0.213 ***	30.96%	[−0.299 ~ −0.139]	Partial	Yes
Model 7: FIRP→AT→PI	H15a	−0.637 ***	−0.387 ***	−0.250 ***	39.25%	[−0.336 ~ −0.174]	Partial	Yes
Model 8: FIRP→SN→PI	H15b	−0.637 ***	−0.387 ***	−0.250 ***	39.25%	[−0.336 ~ −0.174]	Partial	Yes
Model 9: FIRP→PBC→PI	H15c	−0.635 ***	−0.408 ***	−0.227 ***	35.75%	[−0.325 ~ −0.140]	Partial	Yes

Note: *** *p* < 0.001, ** *p* < 0.01. TE—total effects; DE—direct effects; IE—indirect effects; ER—effect ratio.

## Data Availability

Data can be available upon reasonable request.

## Appendix A

**Table A1:** Reliability test results of the pre-survey risk perception scale.

Construct (Cronbach’s Alpha)	Item	Correlation ^a^	Cronbach’s Alpha ^a^
WPRP (0.900)	WPRP1: The source of bottled water may be polluted	0.775	0.871
	WPRP2: The environment around the source of bottled water may be polluted	0.717	0.892
	WPRP3: There may be pollution in the process of bottled water treatment	0.811	0.858
	WPRP4: There may be contamination in the use of bottled water	0.803	0.861
NPPRP (0.896)	PCRP1: Non-degradable packaging of bottled water will cause environmental pollution	0.759	0.869
	PCRP2: Non-degradable packaging of bottled water will affect the surrounding ecological environment	0.754	0.871
	PCRP3: Non-degradable packaging of bottled water will harm humans	0.788	0.858
	PCRP4: Non-degradable packaging of bottled water will cause pollution if it is not recycled properly	0.774	0.864
FIRP (0.924)	FIRP1: Online information about bottled water may have false publicity	0.830	0.902
	FIRP2: Negative information may be hidden in online bottled water publicity	0.854	0.882
	FIRP3: There may be exaggerations in the claimed functions of online bottled water	0.852	0.884

Note: WPRP—water pollution risk perception; NPPRP—non-degradable packaging package pollution risk perception; FIRP—false information risk perception; Correlation ^a^—correlation between the revised item and total score; Cronbach’s alpha ^a^—Cronbach’s alpha after deleting the item.

**Table A2:** Reliability test of the pre-survey planned behavior scale.

Construct (Cronbach’s Alpha)	Item	Correlation ^a^	Cronbach’s Alpha ^a^
AT (0.900)	BA1: Bottled water is safe	0.750	0.880
	BA2: Bottled water is good for your health	0.783	0.869
	BA3: Bottled water is cleaner than tap water	0.734	0.886
	BA4: Bottled water is easy to use	0.843	0.845
SN (0.912)	SN1: People around me approve of my use of bottled water	0.790	0.890
	SN2: People around me support me in using bottled water	0.819	0.880
	SN3: People around me understand that I use bottled water	0.812	0.883
	SN4: People around me recommend that I use bottled water	0.783	0.893
PBC (0.901)	PBC1: I can easily buy bottled water if I want	0.800	0.863
	PBC2: It does not take much time to buy bottled water	0.821	0.845
	PBC3: It does not cost much to buy bottled water	0.793	0.869
PI (0.919)	BI1: I often buy bottled water online	0.856	0.880
	BI2: I will recommend bottled water to others	0.790	0.903
	BI3: I would prefer to buy bottled water online	0.805	0.898
	BI4: I would recommend others to buy bottled water online	0.805	0.898

Note: AT—attitude; SN—subjective norm; PBC—perceived behavior control; PI—purchase intention; Correlation ^a^—correlation between the revised item and total score; Cronbach’s alpha ^a^—Cronbach’s alpha after deleting the item.

**Table A3:** Exploratory factor analysis of the pre-survey perceived risk scale.

Construct	Item	Factor Loading After Direct Rotation Axis of Maximum Variation Method			Commonness
		WPRP	NPPRP	FIRP	
WPRP	WPRP1	0.681	0.413	0.351	0.757
	WPRP2	0.705	0.173	0.477	0.754
	WPRP3	0.832	0.306	0.230	0.839
	WPRP4	0.774	0.423	0.206	0.821
NPPRP	NPPRP1	0.253	0.796	0.248	0.760
	NPPRP2	0.318	0.714	0.346	0.730
	NPPRP3	0.291	0.816	0.223	0.800
	NPPRP4	0.254	0.820	0.176	0.768
FIRP	FIRP1	0.380	0.232	0.806	0.848
	FIRP2	0.230	0.345	0.842	0.880
	FIRP3	0.242	0.232	0.877	0.882
Eigenvalue		2.822	3.181	2.835	8.838
Explain the total variance (%)		25.651	28.918	25.776	80.345
Cumulative explained variance (%)		25.651	54.569	80.345	

**Table A4:** Exploratory factor analysis of the pre-survey planned behavior scale.

Construct	Item	Factor Loading After Direct Rotation Axis of Maximum Variation Method				Commpponness
		AT	SN	PBC	PI	
AT	AT1	0.795	0.278	0.123	0.280	0.803
	AT2	0.824	0.171	0.296	0.128	0.812
	AT3	0.548	0.357	0.455	0.305	0.728
	AT4	0.779	0.251	0.339	0.258	0.851
SN	SN1	0.212	0.786	0.217	0.250	0.771
	SN2	0.134	0.829	0.211	0.265	0.820
	SN3	0.260	0.812	0.213	0.169	0.801
	SN4	0.233	0.797	0.224	0.201	0.780
PBC	PBC1	0.279	0.313	0.765	0.203	0.803
	PBC2	0.272	0.167	0.847	0.211	0.863
	PBC3	0.232	0.310	0.795	0.209	0.826
PI	PI1	0.219	0.254	0.187	0.838	0.850
	PI2	0.180	0.309	0.004	0.842	0.837
	PI3	0.114	0.248	0.334	0.794	0.816
	PI4	0.298	0.095	0.297	0.798	0.823
Eigenvalue		2.789	3.348	2.780	3.265	12.182
Explain the total variance (%)		18.593	22.321	18.532	21.764	81.210
Cumulative explained variance (%)		18.593	40.914	59.446	81.210	
